# Supplementary material for: Sudden Infant Death Associated with Rhinovirus Infection
Source: Viruses. 2024 Mar 27;16(4):518. doi: 10.3390/v16040518 (PMC11054477; doi:10.3390/v16040518)
Supplement: Supplementary file 1 [file viruses-16-00518-s001.zip › viruses-2903432-supplementary.pdf]

Table S1. Primers used in-house PCRs

**A) Primers for EVs and RVs; End-point PCRs for typing and sequencing**		
**Complete VP1, EV-B**		
HEVB-S1695	CTTGTGCTTTGTGTCGGCRTGYAAYGAYTTYTCWG	PCR1
EV2C	CAATACGGCATTTGGACTTGAACTGTATG	
HEVB-S1695	CTTGTGCTTTGTGTCGGCRTGYAAYGAYTTYTCWG	nested PCR
HEVB-R132	GGTGCTCACTAGGAGGTCYCTRTTRTARTCYTCCCA	
P1S1695S	CTTGTGCTTTGTGTCGGC	Sequencing
P2R132S	GGTGCTCACTAGGAGGTC	
**Partial VP1, generic EV**		
224	GCIATGYTIGGIACICAYRT	PCR1
222	CICCIGGIGGIAYRWACAT	
AN89	CCAGCACTGACAGCAGYNGARAYNGG	nested PCR
AN88	TACTGGACCACCTGGNGGNAYRWACAT	
AN232	CCAGCACTGACAGCA	Sequencing
AN233	TACTGGACCACCTGG	
**VP4-VP2, generic RV,**		
RV Savo Sense	GGGACCAACTACTTTGGGTGTCCGTGT	PCR1 and sequencing
RV Savo Rev	GCATC9GGYARYTTCCACCACCANCC	
**B) Primers and probes for gastroenteritis viruses; real time PCRs for detection**		
**Aichi Virus**		
AichiV-FW	CCCAGTGTGCGTAACCTTCT	
AichiV-Rev	GTACCTGCCTGGCATYCCTA	
AichiV-Probe	ABY-ACG CCC TGT GCG GGA TGA AA-QSY	
**Astrovirus**		
ASTV-FW	TCTYATAGACCGYATTATTGG	
ASTV-REV	TCAAATTCTACATCATCACCAA	
ASTV-Probe	FAM-CCCCADCCATCATCATCTTCATCA-QSY	
**Bocavirus**		
HBoV1FW	CCTATATAAGCTGCTGCACTTCCTG	
HBoV234FW	GCACTTCCGCATYTCGTCAG	
HBoV1Rev	AAGCCATAGTAGACTCACCACAAG	
HBoV3Rev	GTGGATTGAAAGCCATAATTTGA	
HBoV24Rev	AGCAGAAAAGGCCATAGTGTCA	
H1234-Probe	FAM-CCAGAGATGTTCACTCGCCG-NFQ-MGB	
**Norovirus, Genogroup I**		
JJV1NFW	CCATGTTCCGTTGGATGC	
JJV1Rev	TCCTTAGACGCCATCATCAT	
JJV1-Probe	ABY-TGTGGACAGGAGATCGCAATCTC-QSY	
RING-1b-Probe	ABY-AGATCGCGGTCTCCTGTCCA-QSY	
**Norovirus, Genogroup II**		

QNIF2dFW	ATGTTCAGRTGGATGAGRTTCTCWGA
COG2Rev	TCGACGCCATCTTCATTCACA
QNIFSProbe	FAM-AGCACGTGGGAGGGCGATCG-QSY
**Rotavirus Group A**	
VP2-F1-fw	TCTGCAGACAGTTGAACCTATTAA
VP2-F2-fw	CAGACACGGTTGAACCCATTAA
VP2-F3-fw	TCGGCTGATACAGTAGAACCTATAAATG
VP2-F4-fw	TGTCAGCTGATACAGTAGAACCTATAAATG
VP2-F5-fw	TCAGCTGACACAGTAGAACCTATAAATG
VP2-R1-rev	GTTGGCGTTTACAGTTCGTTCAT
VP2-R2-rev	GTTGGCGTCTACAATTCGTTCAT
VP2-Probe	FAM-ATGCGCATRTTRTCAAAHGCAA
**Sapovirus**	
SaV124Fw	GAYCASGCTCTCGCYACCTAC
SaV1Fw	TTGGCCCTCGCCACCTAC
SaV1245Rev	CCCTCCATYTCA AACACTA
SaV124TProbe	FAM-CCRCCTATRAACCA-MGB
MS2 phage	inhibition control
MS2-Fw	TGGCACTACCCCTCTCCGTATTCACG
MS2-Rev	GTACGGGCGACCCCACGATGAC
MS2-Probe	VIC-CACATCGATAGATCAAGGTGCCTACAAGC-QSY
An internal control of amplification was added for each PCR, that amplify the bacteriophage MS2	
